# Type-2 diabetes primary prevention program implemented in routine primary care: a process evaluation study

**DOI:** 10.1186/s13063-016-1379-0

**Published:** 2016-05-20

**Authors:** Alvaro Sánchez, Carmen Silvestre, Natalia Campo, Gonzalo Grandes, on behalf of the PreDE research group

**Affiliations:** Primary Care Research Unit of Bizkaia, Basque Health Service (Osakidetza), Bilbao, Spain; Quality Unit, Gipuzkoa Health Region, Osakidetza, Spain; Txagorritxu Hospital, Basque Health Service, Osakidetza, Spain; Primary Care Research Unit of Bizkaia, Luis Power 18, 4a planta, E-48014 Bilbao, Spain

## Abstract

**Background:**

Process evaluation studies are recommended to improve our understanding of underlying mechanisms related to clinicians, patients, context and intervention delivery that may impact on trial or program results and on their potential transferability to practice. This paper aims to document the translation of a type-2 diabetes (T2D) prevention program into the routine context of several primary care centers, assessing process indicators related to clinician adoption, patient recruitment, exposure to the intervention components and baseline characteristics.

**Methods:**

An observational descriptive process evaluation study was conducted of the 2.5-year implementation of the Prevention of Diabetes in Euskadi cluster randomized trial in 14 primary care centers of the Basque Health Service (Osakidetza). The clinical intervention consisted of three components: (1) risk screening, (2) an educational intervention promoting healthy lifestyles, and (3) remote support (follow-up). A passive dissemination strategy of providing training and materials was used to translate the intervention into practice. All non-diabetic patients aged 45 to 70 years who were identified as being at high risk of developing T2D were eligible for study inclusion. The RE-AIM framework guided the process evaluation.

**Results:**

Overall, 31.4 % of family physicians and 57.6 % of nurses participated in the study, while 4170 out of 67,293 (6.2 %) targeted patients who attended the centers during the implementation period were reached through the screening. Around half of the screened patients were identified as being at high risk of developing T2D (FINDRISC score ≥14). The rate of refusal to participate and the proportion of women were higher in the intervention group. Finally, 634 and 454 non-diabetic 45- to 70-year-old patients who were at high risk of T2D were included in the control and intervention group centers (intervention reach = 48 %). Significant variability in most process indicators was observed at center level.

**Conclusion:**

The passive dissemination strategy has produced modest process indicators related to the adoption, reach and implementation of the intervention program, and reduced the possibility of its standardized application in heterogeneous contexts. The resulting different procedures and strategies used by the centers were associated with process outcomes. Context-specific variability and possible confounding will require rigorous procedures for analysis of the intervention effects.

**Trial registration:**

The trial was registered in ClinicalTrials.gov (identifier: NCT01365013). Registered on June 2011.

**Electronic supplementary material:**

The online version of this article (doi:10.1186/s13063-016-1379-0) contains supplementary material, which is available to authorized users.

## Background

Type-2 diabetes (T2D) has become one of the main causes of morbidity and early mortality in most countries. It is expected that its prevalence will double by 2030 and that it will become the seventh leading cause of death worldwide [1]. Therefore, there is a growing interest in the translation of the promising results obtained by several clinical trials in the prevention of T2D through educational interventions to change lifestyles [2–6], into “real-world” clinical practice conditions [7–9]. Unfortunately, this step is not at all easy. The widespread adoption of a promising experimental health intervention does not automatically follow from research proving its efficacy, let alone in the specific context of primary health care (PHC), characterized by work overload, with shortages of time and training. In recent years, there have been several initiatives assessing the transfer of T2D prevention interventions involving the promotion of healthy lifestyles to the real context of PHC [7–10]. However, most of these studies have used non-randomized designs or no comparison groups, were not set up and implemented under real-world conditions of PHC or relied on additional resources, and/or did not include change in the incidence of T2D among the outcome variables. These methodological limitations and mixed results of the majority of the studies raise doubts as to whether the implementation of such programs in normal clinical working conditions can achieve relevant clinical results while maintaining feasibility and sustainability [7].

The translation of evidence-based interventions into routine clinical conditions is a complex challenge and consequently there is a gap between what is known to be effective and what is actually done in the course of routine care in health systems worldwide [11]. Implementation research has emerged in the last decade as a promising way to advance knowledge on how to integrate interventions and treatments of proven efficacy, that are not being widely and sustainably applied, into routine clinical practice. It can be defined as the scientific study of strategies to promote the systematic, widespread, sustainable and continuous adoption of clinical research findings in routine practice. In this growing field of implementation research, one of the important aspects considered in order to ascertain how to enhance the effectiveness, feasibility and sustainability of interventions is their process of implementation [12]. The development, implementation and evaluation of complex health interventions require careful consideration of not only outcomes obtained but also of the processes involved [13, 14].

Process evaluations conducted within trials investigate the implementation, receipt, and setting of an intervention and help in the interpretation of the outcome results and in ascertaining the true implications of interventions in actual practice [14, 15]. Specifically, process evaluation regarding sampling, recruitment, reach and intervention quality facilitate the interpretation of results and help to explain discrepancies between expected and observed outcomes [16]. Process evaluations are especially necessary in multisite trials, where the “same” intervention may be implemented and received in different ways, and consequently may enable us to assess fidelity, monitor intervention doses, and understand how context influences outcomes. This type of process data should be evaluated before the analysis of intervention effects since the results of this evaluation may complete or correct the analysis. On the other hand, a better understanding of program implementation and the barriers and facilitators experienced may also help identify opportunities for optimizing intervention delivery and may inform future implementation and rollout of the intervention in other contexts and settings, and how interventions could move from research to practice. [14, 17]. These other types of process data are usually collected at a later stage [16].

In 2010, the Department of Health of the Government of the Basque Country launched the “Strategy for Tackling the Challenge of Chronicity in the Basque Country” [18]. Among other initiatives, this department commissioned a pilot study of the implementation of a primary program for the prevention of T2D in 14 primary care centers of the Basque Health Service (Osakidetza). Additionally, it funded an independent assessment of the results to provide a basis for future rollout of the program to the other primary care health centers as a new approach to the prevention of T2D in the Basque Country. The Prevention of Diabetes in Euskadi (PreDE) project aims to perform the aforementioned independent evaluation and its primary scientific objective is to assess the effectiveness and feasibility of a T2D prevention intervention involving the promotion of healthy lifestyles in patients who are at high risk of developing the disease, under normal working conditions in primary care in Osakidetza, using an experimental a phase IV clinical trial design [19]. The present study refers to a preliminary process evaluation of the PreDE project. The main objectives of the study are: (1) to document the content of the intervention program actually executed to prevent T2D in the collaborating centers following the use of a classical passive implementation strategy, and to analyze the process indicators of their implementation in the routine context of PHC, and (2) to describe findings related to clinician adoption, patient recruitment, exposure to intervention components and patient baseline characteristics that will help with the interpretation of the PreDE clinical trial results.

## Methods

### Study design and setting

This is an observational descriptive process study of the 2.5-year commissioned implementation of a T2D primary prevention program in 14 PHC centers in Osakidetza that are participating in the PreDE cluster randomized trial (ClinicalTrials.gov NCT01365013). The participating 14 primary care centers were randomly assigned to the intervention or control groups. The PreDE research study was reviewed and approved by the Basque Country Clinical Research Ethics Committee (Ref: 10/2010). Our health service provides universal coverage that is free at the point of use, aside from co-payment for drugs, funded through regional general taxation. Each citizen is included on the list of one family physician or pediatrician who offers comprehensive primary care and constitutes the gatekeeper for referral to hospital services. Primary care professionals work in full-time PHC teams, including family physicians, pediatricians, nurses, and administrative staff based at local centers providing access to health care for users in a defined geographical area.

### Eligibility and recruitment

Non-diabetic patients aged between 45 and 70 years old, who were identified as being at high risk of developing T2D, were eligible for study inclusion in each of the two centers selected by convenience in the seven regions in which the Basque Health Service is organized [19]. The same system for identifying high-risk patients was set up in all the centers, based on the administration of the eight-item Spanish version of the validated Finnish Diabetes Risk Score (FINDRISC) questionnaire [20]. All PHC service users aged between 45 and 70 years who attended one of the participating health centers for any reason during the recruitment period of the program were potential targets for the administration of the FINDRISC questionnaire. Subsequently, all patients detected as having a high risk (FINDRISC score ≥14) were invited to participate and given a patient information sheet. Exclusion criteria for being invited to enroll were doing regular vigorous exercise, or having known diabetes or any other chronic disease that made survival at 6 years unlikely, any condition that could interfere with the metabolism of glucose, or severe cognitive impairment. In all cases, the information sheet explained that they were going to be followed-up for a period of 24 months with annual medical check-ups consisting of blood lipid profile and glucose tolerance tests. In addition, for patients at intervention centers, it provided information on the intervention protocol. Informed consent was obtained from each patient who agreed to participate. Next, they were referred for an oral glucose tolerance test (OGTT) with 75 g of glucose in accordance with the World Health Organization guidelines and assessment of lipid profiles and glycated hemoglobin (HbA1C) levels. Patients who after the test were diagnosed with T2D were seen by their family physician and excluded from the study. At this stage, in the control health centers, high-risk patients with glucose levels below 200 mg/ml in the OGTT at the baseline assessments were included, while in the intervention health centers, patients were included if they obtained these results in the baseline assessments and also agreed to participate in the new educational intervention.

### Intervention standardization

Primary care nursing professionals from the seven intervention centers implemented the DE-PLAN (Diabetes in Europe – Prevention using Lifestyle, Physical Activity and Nutritional-intervention) educational intervention program, while those in the seven control health centers provided usual care for the prevention and treatment of T2D based on the current clinical practice guidelines of Osakidetza. The DE-PLAN program for promoting healthy lifestyles (mainly diet and physical exercise) has two phases: (1) phase 1 consists of intensive intervention through educational sessions in small groups to encourage the abandonment of unhealthy habits and the adoption of healthy habits. Specifically, nurses run four 1.5-hour group sessions. Their objective is to motivate participants to adopt healthy lifestyle habits and to provide information concerning suitable diets and exercise, as well as agree on specific objectives for eating habits and physical activity, (2) phase 2 consists of continuous reinforcement for maintaining motivation through regular contact with participants. Once the intensive education intervention program has been completed, the participants regularly (at least once every 6 weeks) receive reinforcing educational information mainly via telephone calls from nurses through a health communication platform.

### Implementation strategy

The commissioned implementation strategy to adapt the intervention program to the setting and normal working conditions in the health centers in our health service consisted only in classical passive strategies of dissemination such as training and provision of resources (a screening tool embedded in the electronic health record system and educational materials to support educational sessions). The nurses in charge of the intervention group received a 14-hour training course focused on the content and procedures for the educational intervention. Additionally, they received a 5-hour training course on the procedures for screening and identifying patients, and this was also provided to nurses of the control group centers given that the system for screening and identifying patients was the same in both groups.

## Measurements

All data pertaining to recruitment, user demographic characteristics (age, sex, socioeconomic status) and clinical variables (chronic health problems, biological and clinical measures) are derived from data extracted from the electronic health records of Osakidetza. These data were encrypted and managed centrally, together with other measures to safeguard their anonymity and confidentiality, in accordance with the General Law on Data Protection and Patient Autonomy Law of the Spanish Government. Coordination and quality control of the process and execution of the study was the responsibility of the Primary Care Research Unit of Bizkaia in conjunction with the Informatics Department of Osakidetza.

The process and procedures for recruiting eligible patients among those attending the PHC centers was thoroughly assessed and described for both intervention arms, as was the intervention delivery process within the intervention centers. A program intervention matrix was designed for centers to describe the specific procedures (how, when, where), personnel and resources involved for each of the following intervention-related actions: screening of T2D risk among those who attended the center aged between 45 to 70 years, presentation of the informed consent and proposition for participation, baseline clinical testing, annual clinical testing follow-up and educational intervention organizational arrangements and performance. This matrix was sent to and fulfilled by health care professionals before fieldwork period finished and returned to the research team. Doubts were resolved by phone interview with health care professionals.

In order to help the interpretation of process results regarding the impact of the program’s translation to the real-world context of PHC, we used the Reach, Efficacy, Adoption, Implementation, Maintenance (RE-AIM) framework [21] for evaluating the effectiveness and transferability of health behavior programs in terms of public health significance. This framework states that assessment of the public health impact of programs requires more than efficacy testing. Programs must reach a diverse and representative sample of the population at risk. They must be realistic to adopt in specific practice/clinical settings and must be able to be implemented as planned. Lastly, programs must also be maintained over time in a sustainable way by the individual and the practice/clinical setting. Taken together these dimensions determine the overall impact of a program at the population level. Specifically, the indicators for each of the RE-AIM dimensions as they relate to the present process evaluation are:

Adoption (Practice/Center level):

- *A1– Program participation by clinicians*: proportion of family physicians and nurses from the total staff that accepted to participate and have actively collaborated in the program execution.
- Reach (Patient level): exposure of patients to the program at each center:
- *R1– Reach of the screening procedure among potential patients*: proportion of the 45- to 70-year-old non-diabetic patients who attended the center at least once during the program’s recruitment period who had their risk of developing T2D assessed.
- *R2– Reach of the intervention program among eligible patients*: proportion of the 45- to 70-year-old non-diabetic patients attending the center during the program’s recruitment period and identified as having high risk of developing T2D who then received at least one intervention component (included patients).

Additionally, differences in refusal and inclusion rates and characteristics of those exposed to the intervention and control programs were assessed and described at group and center-within-group levels. Patient biological and clinical variables included weight, body mass index, cholesterol, glucose, triglyceride and blood pressure levels, and tobacco use. Level of multimorbidity was characterized in terms of the number of chronic health problems coexisting (from none to four or more) in the same patient, and was assessed by reviewing data for a 4-year period in patient clinical records and applying a list of 52 diseases and specific criteria to consider each disease active, based on previous work related to the burden of chronic conditions in the Basque Country [22–24].

We also evaluated variability in the main process indicators related to program exposure at group and center levels (e.g., study participation refusal rates among those identified as being at high risk) and the impact of recruitment procedures or strategies used on these indicators.

Implementation (Patient level): reception of intervention components as intended:

- *I1*: *Attendance of included patients to intensive educational sessions*. Description of the attendance of included patients (proportion of patients attending) for each of the different educational sessions and formats (group, individual and/or mixed intervention).
- *I2*: *Proportion of included patients completing the intensive educational intervention*. Proportion of included patients who attended all four programmed educational sessions.
- *I3*: *Reception of remote interventions for continuous reinforcement.* Proportion of patients who received remote support, having been contacted at least once or three or more times, mainly through telephone calls or by email.

### Statistical analyses

Frequencies and the proportions were calculated for each of the process indicators. Means and proportions were used to describe patient characteristics for continuous and categorical variables respectively. One-way analysis of variance and chi-squared tests were conducted to determine differences in patient variables at baseline. Logistic regression was used (SAS PROC REG ver. 9.2, SAS Institute, Cary, NC, USA, 2009) to examine the relation of the different implemented strategies and procedures for recruitment and intervention program organization with process indicators related to program exposure. The statistical model for testing the association of implemented strategies and obtained reach included as covariates patient-level variables (age, sex, co-morbidity), and center-level variables (percentage of family physicians and nurses adopting the program), whereas models for the rest of the recruitment process indicators (efficiency of the screening for detecting at risk patients; refusal rate) and for the intervention completion indicator also included body mass index within patient-level variables. In order to account for and test a center variability effect, previous models were extended to generalized random effect models with a random effect at center level (SAS PROC GLIMMIX). This test was also performed to determine and adjust for variability due to center in the group baseline differences in patient variables

## Results

### Adoption among health care professionals

**Fig. 1:**
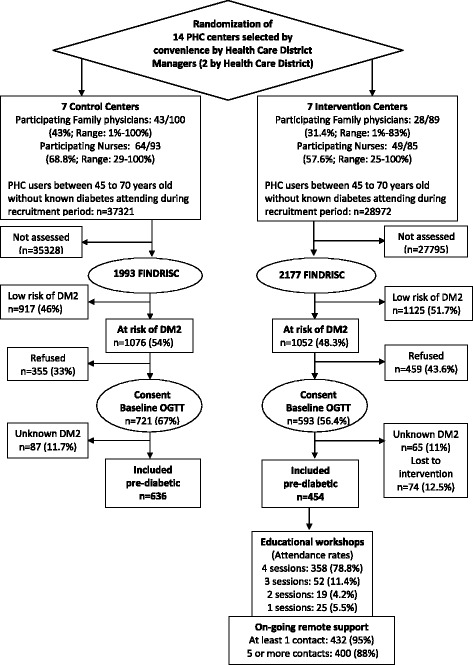
Flow of participant recruitment

Overall, in the seven intervention centers, only 28 from a total of 89 (31.4 %) family physician staff accepted and actively collaborated in the study, while this figure was 49 from a total of 85 (57.6 %) for practice nurse staff (Fig. 1). In the control centers, 43 from 100 of family physician staff and 64 from a total of 93 (68.8 %) practice nurse staff actively collaborated. A high variability in participation rate can be observed at center level. Less than the half of physicians collaborated in four of the intervention centers and in four of the control centers. The lowest participation rate of nurses in the intervention centers was 25 %, yet in five of them more than the half of nurses collaborated.

### Program implementation procedures

Additional file 1 (see Additional file 1. Table outlining the procedures and strategies used by each center) sets out the specific procedures employed in centers for each intervention-related action (screening of T2D risk, informed consent and invitation to participate, clinical tests, organizational arrangements and delivery of the educational intervention). Overall, screening of T2D risk among potential patients was mainly performed by collaborating nurses in routine or programed consultations. In some of the centers, family physicians occasionally collaborated in the organization of screening procedures. Other procedures used with the intention of increasing screening rates were assessing patients when they attended the center for other activities (wound care or tests), placing posters and paper-based screening questionnaires in the center’s waiting rooms, and organizing 1-day public screening in the street. In some centers, a more specific strategy was agreed for selecting potential patients for screening (e.g., those having an additional risk factor such as obesity).

In the case of a patient being identified as high risk of developing T2D, most of the collaborating centers invited them to participate in the study and gave them informed consent forms directly after completing the screening. In contrast, some centers gave patients more time to reflect and invited them for an additional consultation in which they were asked whether they agreed to participate. Baseline clinical testing was mostly performed in collaborating centers, but with variations in the schedule offered (1 day versus various days). Four of the centers were unable to offer the blood tests and/or OGTTs and hence participants were sent to the referral hospital. Feedback on clinical testing was given over the telephone or during in-person appointments with the nurse or the family physician. The annual follow-up clinical testing was performed on 2 days in most of the centers, with OGTTs and other blood tests on one day and the rest of clinical measurements (weight, blood pressure, etc.) on another.

Within the centers allocated to the intervention group, organizational arrangements for the intervention were mainly performed by collaborating nurses who, after agreeing on a timetable for the educational workshop sessions, contacted patients by telephone. Patient preferences on the timing of sessions were only taken into account in one of the centers. Regarding timetable and format, most of the centers offered patients the possibility of attending in the morning or the afternoon and a group format, but with the option of individual sessions on request. Two centers offered only morning or only afternoon workshops, while one of the centers offered only group-format workshops. All centers except one ran the educational sessions in the center itself.

### Recruitment of patients among groups

Figure 1 and Table 1 present the flow diagram and the process indicators of the recruitment and intervention delivery process. In short, from the 67,293 target patients aged 45 to 70 years who attended the centers during the recruitment period, a total of 4170 patients were approached through the screening of risk for T2D (FINDRISC). Within the comparison groups, 1993 from 37,321 and 2177 from 29,972 were approached in the control and intervention centers respectively. Thus, the reach of this screening at the potential target population level in routine context of PC (R1) was 5.3 % in the control group and 7.3 % in the intervention group. Of those approached, 1076 (54 %) and 1052 (48.3 %) patients were identified as being at high risk of developing T2D (FINDRISC ≥14 points). When presented with the project information sheets and informed consent forms, 355 (33 %) and 459 (43.6 %) of the high-risk patients refused to participate in the study while 721 and 593 were referred for baseline OGTTs and clinical assessment in the control and intervention centers respectively. This translates into an absolute between-group difference in participation rate of 10 %. In the statistical analysis of the significance of this difference, considering the clustered nature of data of participants within centers and a between-cluster randomization design, the parameter estimating the effect of the group was not significant (*p* = 0.27), while the hypothesis test regarding the significance of the “center” random effect was highly significant (*p* <0.001).

**Table 1:** Variability in patient recruitment process indicators among comparison groups and collaborating centers

PHC center	Months recruiting	Attendees	FINDRISC	Screening reach	At risk of T2D	OGTT performed	Refusal rate	Non- diabetic	Included in study
		*n* Total	*n* Total		*n* (%)	*n*		*n* (%)	*n*
Intervention centers									
SV	24.5	4097	256	6.2 %	158 (61.7 %)	114	27.8 %	100 (87.7 %)	77
GK	25.7	7440	282	3.8 %	212 (75.2 %)	116	44.3 %	98 (84.5 %)	82
BL	20.8	2068	296	14.3 %	116 (39.2 %)	52	55.2 %	47 (90.4 %)	39
LG	18.6	1951	391	20.0 %	125 (32.0 %)	57	54.4 %	52 (91.2 %)	51
DB	28.2	6229	125	2.0 %	100 (80.0 %)	59	41.0 %	56 (94.9 %)	54
IZ	26.1	5106	456	8.9 %	260 (57.0 %)	162	37.7 %	143 (88.3 %)	123
AR	17.6	3081	371	12.0 %	81 (21.8 %)	33	59.2 %	32 (96.7 %)	28
Total		29,972	2177	7.3 %	1052 (48.4 %)	593	43.3 %	528 (88.4 %)	454
Control centers									
ZU	28.6	4632	310	6.7 %	219 (70.6 %)	176	19.6 %	146 (83.0 %)	146
LL	25.4	5041	256	5.1 %	150 (58.6 %)	106	28.7 %	93 (87.7 %)	93
TX	19.4	3928	509	13.0 %	165 (32.4 %)	82	43.0 %	78 (95.1 %)	78
AZ	25.8	3370	325	9.6 %	205 (63.1 %)	163	19.0 %	143 (87.7 %)	143
IR	14.9	6227	128	2.1 %	79 (61.7 %)	41	45.6 %	35 (85.4 %)	35
AB	22.7	5589	183	3.3 %	138 (75.4 %)	100	26.8 %	90 (90.0 %)	90
SM	26.3	8534	282	3.3 %	120 (42.6 %)	53	54.2 %	49 (92.4 %)	49
Total		37,321	1993	5.3 %	1076 (54.0 %)	721	33.0 %	634 (88.3 %)	634

*T2D* type-2 diabetes, *FINDRISC* Finnish Diabetes Risk Score, *OGTT* oral glucose tolerance test, *PHC* primary health care

After performance of the baseline OGTT, 87 (11.7 %) and 65 (10.9 %) of patients were diagnosed with unknown diabetes with glucose levels of ≥200 mg/ml and excluded from the study. Therefore, 634 45- to 70-year-old non-diabetic patients who were at high risk of developing T2D were included in the control group centers, while in the intervention arm, 454 patients were finally included, as 74 patients despite consenting did not actually participate in the intervention due to difficulties attending the sessions. Thus, after removing the proportion of diagnosed unknown diabetes (11 %) from those detected as being at high risk (*n* = 1052), the estimated reach of the intervention program to eligible 45- to 70-year-old non-diabetic patients who were at high risk of developing T2D (R2) was 48 %.

### Characteristics of included patients among groups and centers within groups

As described in Table 2, included patients had a mean age of 59 years, and almost two thirds were women (62.3 %). More than a half were obese (55.5 %) and had two or more chronic diseases (52.4 %), while 13 % were smokers. All measured personal and clinical characteristics were balanced between groups except for sex with a higher percentage of included patients being women in the intervention group (66.6 % versus 58 % in the control group; *P* = 0.05, controlling for center variability). A notable variability was evidenced at center level: the percentage of included patients who were women ranged from 48.6 to 69.7 % and from 51.2 to 81.1 % in collaborating centers in the control and intervention groups (gender distribution in collaborating centers not shown) respectively, this center variability being highly significant (*p* <0.003).

**Table 2:** Baseline characteristics of included patients

		Control (*n* = 636)	DE-PLAN (*n* = 454)
Age		59.3 (6.9)	59.3 (6.9)
% women		58.0 %	66.5 %
Weight, kg		82.9 (15.2)	80.8 (14.8)
Body mass index, kg/m^2^		31.0 (4.7)	31.0 (5.0)
% overweight		38.0 %	35.0 %
% obese		55.4 %	56.0 %
Cholesterol levels, mg/dL		213.7 (34.7)	217.0 (34.5)
Triglyceride levels, mg/dL		124.5 (65.9)	122.7 (60.6)
Glucose levels, mg/dL		104.6 (11.8)	104.5 (12.4)
Systolic blood pressure, mm/Hg		134.7 (15.5)	132.5 (14.3)
Diastolic blood pressure, mm/Hg		80.9 (9.6)	80.4 (8.8)
% elevated blood pressure		10.5 %	10.8 %
% smokers		13.2 %	12.6 %
Co-morbidity (%)			
	0	16.6 %	15.7 %
	1	32.7 %	30.3 %
	2	24.4 %	25 %
	3	14.2 %	13.7 %
	4 or more	12.1 %	15.3 %

*DE-PLAN* Diabetes in Europe – Prevention using Lifestyle, Physical Activity and Nutritional-intervention

### Variability in program implementation indicators at center level

The main recruitment process indicators varied among collaborating centers (test for the random effect, *p* <0.001). Screening reach ranged from 2.1 to 13 % in the control group, and from 2 to 20 % in the intervention group. There was also considerable variation in the efficiency of the screening procedures at finding individuals who were at high risk of developing DT2, the percentage of patients found to be at risk out of all those assessed ranging from 21.8 to 80 % in the intervention group, and from 32.4 to 70.6 % in the control group. As previously mentioned, refusal rate varied significantly across the collaborating health centers (*p* <0.001): the lowest and highest refusal rates were 27.8 % and 58 % in the intervention group, and 19 % and 54.2 % in the control group.

### Impact of employed procedures on process indicators

Table 3 displays the associations of strategies carried out by centers to execute the program components and the main process indicators related to recruitment and intervention completion. Centers that used massive screening strategies, namely, assessing potentially at-risk patients attending the procedure/wound care room or for diagnostic/clinical tests, or placing posters and questionnaires in corridors and waiting rooms, tended to obtain a higher reach but lower rates of efficiency in detecting high-risk patients. In contrast, those centers that employed a more specific strategy for selecting potential patients for screening (i.e., those having an additional risk factor such as obesity) had a lower reach but higher efficiency rates for detecting at-risk patients out of the total assessed. After controlling for covariates at patient (sex, age, co-morbidity) and center (percentage of collaborating family physicians and nurses, center as a random effect) levels, implementing a more specific screening strategy was significantly associated with obtaining lower reach rates (OR = 0.36; 95 % CI: 0.11 to 0.86), and with a higher likelihood of obtaining a positive risk test among patients assessed, this being 3.2-fold higher (95 % CI for OR = 2.08 to 4.93).

**Table 3:** One-way adjusted associations between implemented program procedures and strategies, and recruitment and intervention completion process indicators

Variable	Reach of the screening^a^	Efficiency of the screening^b^	Refusal rate^b^	Completion of intervention^b^
Massive screening strategies	0.69 (0.19–2.50)	0.57 (0.26–1.23)	2.34 (1.08–5.07)	N.A.
Targeted screening strategy	0.36 (0.11–0.86)	3.2 (2.08–4.93)	0.29 (0.18–0.47)	N.A.
Additional appointment for informed consent	N.A.	N.A.	3.34 (1.36–8.21)	N.A.
Baseline test offered on only one specific day	N.A.	N.A.	0.73 (0.08–6.59)	N.A.
Blood test out of center	N.A.	N.A.	2.15 (0.81–5.75)	N.A.
Intervention offered at one time of day only	N.A.	N.A.	N.A.	0.72 (0.40–1.30)

Values are odds ratios (95 % confidence interval); *N.A*. not applicable, ^a^Model 1: adjusted for patient-level variables (age, sex, co-morbidity) and center-level variables (percentage of family physicians and nurses adopting the program) and center random effect, ^b^Model 2: same adjustment as ^a^, but including body mass index within patient-level variables. Center random effect in all tested models *p* <0.001

Three of the strategies used were associated with refusal rates. Refusal to participate in the study was twice as likely in centers that used massive screening strategies (OR = 2.34; 95 % CI: 1.08 to 5.07). Higher rates of refusal to participate were also more frequent in centers in which patients had to attend an additional consultation to communicate their decision on whether to participate and provide written informed consent (OR = 3.34; 95 % CI: 1.36 to 8.21) compared to those in centers where patients were given the informed consent form on the same day as the screening test. In contrast, a lower refusal rate was associated with use of targeted screening based in additional risk factors (OR = 0.29; 95 % CI: 0.18 to 0.47). The effect of other strategies (e.g., blood tests being performed outside the center) was not significantly associated with refusal rate beyond the aforementioned significant variability at center level.

### Implementation of the intervention at patient level

As described in Table 4, 454 from a total of 528 eligible patients (45- to 70-year-old non-diabetics who were at high risk of developing T2D) initiated the intervention, translating to a reach of the intervention program of 48 %. Regarding the format on the received intervention, the majority of intervention patients (*n* = 418) selected the group format, while 28 and 8 chose individual or mixed formats respectively. Out of all included patients, 358 (78.8 % of those included in the intervention group) completed the intervention program, attending all of the scheduled workshop sessions (330 group format; 20 individual format; 8 mixed format). At least three sessions were completed by 52 patients (11.3 %), while 19 patients (4.1 %) completed two sessions and 25 (5.7 %) completed only one session. Ongoing remote support via telephone calls or emails was received by 91 % of intervention patients, with 85.8 % of them receiving five or more contacts. Lastly, regarding the organization of the workshop sessions, offering only one timing, morning or evening, was not associated with intervention completing rate (OR = 0.72; 95 % CI: 0.40 to 1.30), after controlling for patient-level covariates and “center” random effect as a source of variation.

**Table 4:** Variability in intervention-related process indicators among collaborating centers allocated to the intervention group

PHC center	Intervention reach^a^	Educational sessions completed	Intervention format (% group, individual, mixed)	Reception of ≥5 remote support contacts
SV	54,7 %	96.1 %	98.7 %, 1.3 %, 0 %	94.8 %
GK	44,7 %	80.9 %	100 %, 0 %, 0 %	79.8 %
BL	36,8 %	82.5 %	89.7 %, 7.7 %, 2.6 %	77.5 %
LG	44,4 %	92.2 %	62.7 %, 37.3 %, 0 %	92.2 %
DB	56,8 %	64.8 %	90.7 %, 5.6 %, 3.7 %	81.5 %
IZ	52,8 %	66.7 %	100 %, 0 %, 0 %	86.2 %
AR	35,6 %	75.9 %	75 %, 1 %, 17.9 %	86.2 %
Total	47,9 %	78.8 %	92.1 %, 6.2 %, 1 .8 %	85.8 %

^a^Calculated as the proportion of patients included in the intervention from those detected as being at high risk of T2D after removing the proportion of diagnosed unknown T2D patients in each of the centers

## Discussion

The present study aimed to evaluate the process indicators regarding the commissioned implementation of a T2D primary prevention intervention program in routine conditions of PHC, in order to facilitate subsequent analysis of effects and their interpretation and to inform future optimization of the implementation in clinical practice. In short, the main results of this process evaluation showed that the implemented strategy, primarily based on classical passive strategies of dissemination such as training and provision of resources, attained modest-to-good process indicators related to adoption, reach, and implementation of the intervention program. However, there was evidence of a notable variability in process indicators by center.

As underlined by the RE-AIM framework, one of the major factors determining the potential impact of programs is that they must be appealing to health care providers and realistic to implement in practice settings. The literature points to competing demands and lack of time as the main reasons for refusal to participate among PHC professionals [25]. In the present study, rates of adoption of clinicians within centers were fair but improvable, especially among family physicians. In five out of seven centers in each of the comparison groups, the program adoption rate has exceeded 50 % among nurses, and only in two centers in each of the groups was the level of collaboration similarly high among family physicians. We are unable to compare these adoption rates with the findings of other studies on the translation of diabetes prevention programs as they do not report these types of data regarding the rate of adoption among health care professionals.

Overall, the reach of the screening procedures to identify individuals at risk of developing T2D among 45- to 70-year-old PHC users was 6.2 % (range 2 to 20 %).

Though these figures could be considered low, they must be considered valuable as the program has been conducted in the real context of primary health care delivery process without altering usual working conditions. All patients of that age strata who attended the centers due to any possible health complication were potential participants and were addressed by professionals after resolving the problem that caused their attendance. Other studies aimed at translating a T2D primary prevention program to PHC have attained higher reach indicators ranging from 44 to 56 %, but after conducting a previous selection of patients from electronic health records and contacting them by letter or telephone [26–28]. The present study covered a much larger and broader target population than similar translation studies of T2D primary prevention in PHC, without a previous selection procedure of eligible patients being carried out, thus better reproducing the real-world conditions of PHC services. Nevertheless, between-center variability in attained reach shows that figures could be improved and this warrants further investigation on how to achieve a higher but tolerable reach.

Around half of the 45- to 70-year-old patients attending PHC who were assessed were detected as having a high risk of developing T2D (FINDRISC score ≥14), a figure within the range previously reported in the literature [29]. However, as occurred with the reach of the screening there was substantial within-group center variability in the efficiency in detecting high-risk patients. Though all centers were instructed to approach patients and screen considering only patient age (i.e., “all patients attending the health center aged between 45 and 70 years”), some centers targeted the screening to high-risk patients, while others employed more universal strategies. These different procedures affected both reach rates and efficiency in detecting individuals who were at high risk of developing T2D: the more massive screening strategies had higher reach among attending users but lower percentages of detection of high-risk patients, the opposite occurring in centers that restricted the screening strategy by only assessing those with additional risk factors.

Not all the patients detected as having a high risk of developing T2D were finally included in the study. The research-related operational and ethical requirements, and the real-world context of PHC together with an evidenced variability in procedures and strategies adopted between centers, have determined two major issues that could affect future analysis and interpretation of the program’s effects: differences in refusal rates and in baseline comparability among comparison groups. First, a higher rate of refusal to participate in the study was encountered by collaborating clinicians in the intervention group centers. This may be attributable to the fact that these patients were not only asked to consent to the annual testing but also were invited to attend four educational workshop sessions. Second, after exclusion of a considerable proportion of undiagnosed cases of T2D (approximately 12 % in both groups), a higher proportion of women were included in the intervention group, pointing to a possible selection bias. These two sources of potential confounding would threaten the internal validity of a phase III clinical trial under controlled conditions [30]. In the case of a phase IV implementation trial, aiming to conduct a broad evaluation of the translation of proven efficacy interventions into routine care, the focus should be on external validity and generalizability, thus assessing effectiveness in heterogeneous, unselected populations and real-world clinical settings [30, 31]. In any case, and taking the worst case scenario of analyzing this clustered trial as an observational study, rigorous analytic procedures will need to be used in the analysis of effects of this intervention in order to reduce and deal with the possible confounding and context-specific variability [32].

When analyzing the source of observed differences in refusal rates and in the proportion of women included, three important factors must be noted. First, as previously observed with the screening reach and its efficiency at detecting high-risk patients, refusal rate is associated with procedures and strategies for recruitment. Specifically, centers that used massive screening strategies and those that did not obtain patient consent to participate directly after the screening (offering an additional consultation) had higher odds of refusal. Second, strong between-center variability was present even after adjusting for the effect of group, these differences between centers being at least as relevant as those observed at group level for explaining differences in both refusal rates and percentage of women included. Lastly, considering that a passive implementation strategy reduces the possibility of a standardized implementation of programs, leaving the responsibility for program organization decision-making to health professionals themselves, this observed variability at center level could be expected. Variability in program adoption by professionals among different sites and in program reach and implementation indicators have also been reported in other implementation trials [33, 34]. Every center has a different context and thus implementation strategies must be targeted and adapted to that specific context. Even after a hypothetical adaptation, results and products would be context specific as changes are common when implementing interventions in practice settings [35]. Therefore, a lesson learned from the current process evaluation is that context is crucial, this meaning that, when using passive strategies for implementation, an a priori standardized intervention would be implemented in different ways in different settings resulting in different process and possibly clinical outcomes [33, 36].

Evidence has clearly demonstrated that T2D can be prevented or delayed in at-risk individuals through intensive healthy lifestyle counseling [7–10]. The reach of the intervention program of 48 % out of all the eligible pre-diabetic patients identified can be considered as moderate [37]. Moreover, the quality of the intervention program implementation can be considered good, as more than 80 % of the patients in the intervention group finally included received all the components of the educational intervention program. This overall program attendance rate is within the range of other diabetes primary prevention programs in PHC [8, 37, 38]. Again, program completion rates varied between intervention centers but it was not associated with the flexibility in the schedules and formats offered by centers.

To the best of our knowledge, this is the first study assessing the main process indicators of a translation of a T2D prevention program in the routine PHC context. However, the present study has several limitations. First, the process evaluation refers to 14 selected centers. Although collaborating centers seem to be quite diverse, they may not be representative of all PHC centers across our health service. In addition, though we have described some characteristics regarding representativeness, in terms of PHC size and composition, socio-economic status of attended populations, and so on, a lack of some other types of information regarding specific context characteristics may limit the interpretation of results. Though the external validity of the study could be questioned, implementation trials aim to conduct a broad evaluation of the translation of proven efficacy interventions into routine care, thus assessing results in heterogeneous, unselected populations and real-world clinical settings [30, 31]. Another limitation of the present study is that it is mainly based on quantitative data. Ascertaining the factors behind implementation heterogeneity from a qualitative perspective might help in the interpretation of results.

## Conclusion

To determine whether a health promotion program is feasible when translated into the real-world clinical setting, important information regarding the main factors that affect external validity should be provided. The present process evaluation study reports on the adoption, reach, implementation and other process indicators attained by a T2D primary prevention program rolled out in PHC to determine its actual feasibility. The modest rates of adoption by clinicians and reach of the potential population reflect the difficulties of integrating a new intervention within the established functioning of care delivery in PHC. Moreover, observed variability of indicators at health care context level also points to the need to adapt the intervention components and their operation to handle the competing demands of routine health care services. A strong theoretically grounded implementation strategy to enhance the adoption and execution of an evidence-based clinical intervention, rather than the classical passive dissemination strategies used within the present commissioned implementation, might obtain better outcomes.

## Additional file

Additional file 1: Table outlining the procedures and strategies used by each center. (DOCX 19 kb)

## Abbreviations

CI: confidence interval
DE-PLAN: Diabetes in Europe – Prevention using Lifestyle, physical Activity and Nutritional-intervention
FINDRISC: Finnish Diabetes Risk Score
OGTT: oral glucose tolerance test
OR: odds ratio
PHC: primary health care
PreDE: Prevention of Diabetes in Euskadi
RE-AIM: Reach, Efficacy, Adoption, Implementation, Maintenance framework
T2D: type-2 diabetes
