# Supplementary material for: Type-2 diabetes primary prevention program implemented in routine primary care: a process evaluation study
Source: Trials. 2016 May 20;17:254. doi: 10.1186/s13063-016-1379-0 (PMC4875717; doi:10.1186/s13063-016-1379-0)
Supplement: Additional file 1: — Table outlining the procedures and strategies used by each center. (DOCX 19 kb) [file 13063_2016_1379_MOESM1_ESM.docx]

**Additional file 1. Table outlining the procedures and strategies used by each centers**

|  |  | **Intervention Centers** | | | | | | | **Control Centers** | | | | | | |
| --- | --- | --- | --- | --- | --- | --- | --- | --- | --- | --- | --- | --- | --- | --- | --- |
| **Action** | **Procedure** | **Ara** | **Lgz** | **Iztie** | **Gal** | **Bol** | **Dub** | **SVic** | **AB** | **Azp** | **Irun** | **Llod** | **SM** | **Txrd** | **Zz** |
| **1. Screening for risk of developing T2D (FINDRISC) in patients attending the center age 45 to 70 years** | In appointments with nurses | X | X | X | X | X | X | X | X | X | X | X | X | X | X |
|  | Additionally, in appointments with doctors | X | X |  | X | X | X |  |  | X |  |  |  |  | X |
|  | Additionally, when attending for tests or other procedures or wound care |  | X | X |  |  | X |  |  |  |  |  |  |  |  |
|  | Posters and/or questionnaires in waiting rooms at the center | X | X | X |  |  |  |  |  |  |  |  | X | X |  |
|  | One-day of screening in the street outside the center |  | X | X |  |  |  |  |  |  |  |  |  |  |  |
|  | More specific targeting based on risk factors (obesity, high blood pressure, etc.) | X |  |  |  |  | X | X | X | X |  |  |  |  | X |
| **2. Request for informed consent for participation from at-risk patients (FINDRISC ≥ 14)** | The same day as the screening, by the nurse or the doctor | X | X | X | X |  | X | X | X | X |  | X |  | X | X |
|  | In a dedicated extra appointment with the nurse |  | X |  | X | X | X | X |  |  | X |  | X | X |  |
| **3. Baseline tests and measurements** |  |  |  |  |  |  |  |  |  |  |  |  |  |  |  |
| 3.a. Request for tests and measurements | The same day as the screening, by the nurse or the doctor | X | X | X | X |  | X | X | X |  |  | X |  | X | X |
|  | In an extra appointment made at the time of the screening |  | X |  |  | X |  | X |  | X | X |  | X | X |  |
| 3.b. Performance of tests and measurements | In the health center  on one day of the week | X |  |  | X |  |  |  |  |  |  |  |  |  |  |
|  | In the health center on various days of the week |  | X | X |  |  |  | X | X | X |  | X |  | X | X |
|  | In the referral hospital on one day of the week |  | X |  |  |  | X |  |  |  | X |  |  | X |  |
|  | In the referral hospital on various days of the week |  |  |  |  | X |  |  |  |  |  |  | X |  |  |
| 3.c. Delivery of results | Over the telephone by a nurse |  | X | X | X | X | X |  |  |  |  |  |  |  |  |
|  | Over the telephone by a doctor |  |  |  |  |  |  |  |  |  |  | X |  | X |  |
|  | Over the telephone by a member of the administrative staff | X |  |  |  |  |  |  |  |  |  |  |  |  |  |
|  | During an appointment with a doctor |  |  |  |  |  |  | X | X | X |  |  |  |  | X |
|  | During an appointment with a nurse |  |  |  |  |  |  |  |  |  | X |  | X |  |  |
| **4. Annual follow-up (tests and clinical assessments)** | All in a single scheduled appointment | X |  |  | X | X |  |  |  | X |  |  |  |  |  |
|  | Two appointments, one for blood tests and one for other measurements |  |  | X |  |  | X | X | X |  | X | X | X | X | X |
|  | Monthly appointments in person for measurements and weight education |  |  |  |  |  | X |  |  |  |  |  |  |  |  |
| **5. Educational intervention** |  |  |  |  |  |  |  |  |  |  |  |  |  |  |  |
| 5.a. Organization of the workshop sessions | Participants informed of dates and times of sessions once agreed between staff involved | X | X | X | X | X | X | X |  |  |  |  |  |  |  |
|  | Participants contacted to ask their preferences about timings of sessions |  | X |  |  |  |  |  |  |  |  |  |  |  |  |
| 5.b. Delivery of the sessions | In the center |  | X | X | X | X | X | X |  |  |  |  |  |  |  |
|  | Outside the center | X |  |  |  |  |  |  |  |  |  |  |  |  |  |
|  | Morning and afternoon sessions offered |  | X | X | X | X |  | X |  |  |  |  |  |  |  |
|  | Only morning sessions | X |  |  |  |  |  |  |  |  |  |  |  |  |  |
|  | Only afternoon sessions |  |  |  |  |  | X |  |  |  |  |  |  |  |  |
|  | Both individual and group sessions | X | X | X |  | X | X | X |  |  |  |  |  |  |  |
|  | Group sessions only |  |  |  | X |  |  |  |  |  |  |  |  |  |  |
